# Exploring Neuroprotective Agents for Sepsis-Associated Encephalopathy: A Comprehensive Review

**DOI:** 10.3390/ijms241310780

**Published:** 2023-06-28

**Authors:** Klaudia Krzyzaniak, Robert Krion, Aleksandra Szymczyk, Ewelina Stepniewska, Mariusz Sieminski

**Affiliations:** Department of Emergency Medicine, Medical University of Gdansk, Smoluchowskiego 17, 80-214 Gdansk, Poland; klaudiak@gumed.edu.pl (K.K.);

**Keywords:** sepsis associated encephalopathy, SAE, sepsis, neuroprotection

## Abstract

Sepsis is a life-threatening condition resulting from an inflammatory overreaction that is induced by an infectious factor, which leads to multi-organ failure. Sepsis-associated encephalopathy (SAE) is a common complication of sepsis that can lead to acute cognitive and consciousness disorders, and no strict diagnostic criteria have been created for the complication thus far. The etiopathology of SAE is not fully understood, but plausible mechanisms include neuroinflammation, blood–brain barrier disruption, altered cerebral microcirculation, alterations in neurotransmission, changes in calcium homeostasis, and oxidative stress. SAE may also lead to long-term consequences such as dementia and post-traumatic stress disorder. This review aims to provide a comprehensive summary of substances with neuroprotective properties that have the potential to offer neuroprotection in the treatment of SAE. An extensive literature search was conducted, extracting 71 articles that cover a range of substances, including plant-derived drugs, peptides, monoclonal antibodies, and other commonly used drugs. This review may provide valuable insights for clinicians and researchers working in the field of sepsis and SAE and contribute to the development of new treatment options for this challenging condition.

## 1. Introduction

Sepsis is defined as a syndrome of physiological, pathological, and biochemical abnormalities, and it results from an inflammatory overreaction that is induced by an infectious factor, which leads to multi-organ failure in some cases. The most common starting point of the infection is the abdominal cavity, after which the infection spreads throughout the body. A diagnosis of sepsis is performed based on the patient achieving two or more points on the Sequential Organ Failure Assessment (SOFA) scale [1]. The global incidence of sepsis is approximately 20 million cases per year; the mortality rate is estimated to be 5%, but in cases of septic shock, it can reach 50% [2,3]. Among hospitalized patients, the mortality rate is approximately 27%, making sepsis one of the leading causes of in-hospital mortality [4]. Most cases of severe sepsis that end with death occur in intensive care units (ICUs) due to the serious conditions of the patients who are exposed to hospital infections [5]. Hyperacute therapeutic intervention after a diagnosis of sepsis is recommended, including an aggressive supply of crystalloids, intravenous supply of vasopressors, and antibiotic therapy in the first hour after septic diagnosis [6].

Sepsis-associated encephalopathy (SAE) is a complication of sepsis that most commonly manifests in the form of acute cognitive and consciousness disorders [Table 1]. There are a vast range of SAE symptoms. Some of these are discrete, such as confusion, depression, agitation, or sedation, which can make SAE easy to miss. The more severe symptoms include delirium, seizures, flapping tremors, paratonic rigidity, and even coma in the most severe cases [7]. It is worth noting that, to date, no strict diagnostic criteria for SAE have been created. Researchers focusing on this topic define it as an acute worsening of a patient’s cognitive competencies and an acutely decreased level of consciousness that is related to the presence of sepsis. SAE increases the 30-day mortality rate of patients with sepsis up to 60% [8,9]. Trustworthy data on the prevalence of SAE are inconclusive, and there have been predictions that the statistics lie between 9% and 70% for patients with severe sepsis. Specifically, SAE’s incidence is high among patients that present organ failure [7,10].

The etiopathology of SAE is not fully understood, and there are several plausible mechanisms: neuroinflammation, blood–brain barrier (BBB) disruption, altered cerebral microcirculation, alterations in neurotransmission, changes in calcium homeostasis, and oxidative stress. Proinflammatory cytokines can diffuse to the brain via a disrupted blood–brain barrier and stimulate microglia to produce more cytokines, exacerbating inflammation. In addition, activated endothelial cells produce reactive oxygen species that lead to the apoptosis of neurons. In terms of alterations in neurotransmission, the most commonly described ones are dysregulation in the cholinergic pathway, which results in a lack of acetylcholine and causes symptoms that are similar to delirium. Different neurotransmitters such as norepinephrine, dopamine, serotonin, and gamma-aminobutyric acid are also being researched [11,12,13]. The data on altered cerebral circulation are inconsistent. In studies on septic animal models, no changes in brain circulation were observed; on the other hand, research on human subjects has indicated that the cerebral autoregulation of circulation is impaired [14,15].

Sepsis-associated encephalopathy may lead to long-term consequences, with dementia being at the forefront, as well as symptoms that are similar to those associated with Alzheimer’s disease. A reduction in the volume of the hippocampus and long-term changes in EEG results were observed in some of the survivors [16]. Patients presented deficits in memory and learning capabilities [17]. Post-traumatic stress disorder was diagnosed in 12% of patients six months after hospitalization due to severe sepsis; unfortunately, there was no information recorded regarding the presence of SAE symptoms [18].

To achieve the objective of this review, an extensive literature search was conducted, which focused on relevant terms such as “neuroprotection in SAE” and “neuroprotection in sepsis”. The resulting articles were carefully evaluated to identify those that offered insight into the use of substances with neuroprotective properties for the treatment of SAE. After eliminating articles with unrelated topics, 42 articles remained. Further searches were conducted to discover studies related to the substances in question. Finally, 71 articles were selected.

Through this review, we hope to provide a valuable resource for clinicians and researchers working in the field of sepsis and SAE, and we hope to contribute to the development of new treatment options for this challenging condition. This review aims to present an overview of the current state of knowledge on this topic, including the substances that have been studied for their neuroprotective effects, the mechanisms by which they exert their actions, and the results of clinical trials and preclinical studies. The selected articles cover a range of substances, including plant-derived drugs, peptides, monoclonal antibodies, and other commonly used drugs.

## 2. Sedative Drugs

Published studies point to the neuroprotective roles of the drugs that are routinely used in the ICU and during surgical procedures. Dexmedetomidine is one drug that is commonly used for the mild sedation of patients. It is a highly selective alpha-2 adrenoceptor agonist, and it is able to diffuse across the blood–brain barrier and ameliorate the symptoms of SAE by acting on alpha-2 adrenoceptors in astrocytes [19]. Sevoflurane and isoflurane, which are anesthetic gases used in general anesthesia, increase GABA and potassium channel activity; decrease acetylcholine, dopamine, and serotonin neurotransmission; and reduce blood pressure [20].

### 2.1. Human Studies

In one study, patients treated with dexmedetomidine had a lower risk of delirium and coma. Compared with lorazepam, dexmedetomidine reduced the 28-day mortality rate (17 vs. 27%) [21]. However, another study showed that there were no differences in the mortality rate between groups of patients aged over 65 years who had been treated with dexmedetomidine and those who did not receive the treatment [22].

### 2.2. Animal Studies

Animal models were obtained using cecal ligation and puncture (CLP). After CLP, mice treated with dexmedetomidine had better results in Morris water maze (MWM), Barnes maze, and fear conditioning tests, as well as reduced concentration of proinflammatory cytokines in the hippocampus, polarization of T lymphocytes, and reduced blood–brain barrier interruption [19]. These results suggest that dexmedetomidine reduces the inflammatory response in the acute phase and, moreover, protects animals against cognitive and behavioral deterioration. In addition, it has been shown that dexmedetomidine affects the Hsp90/AKT pathway, which plays a regulatory role in the survival and apoptosis of cells, resulting in decreased hippocampal cell mortality [23]. The dexmedetomidine treatment of sepsis induced by an intraperitoneal injection of lipopolysaccharide led to a reduction in brain inflammation and oxidative stress. The treated animals had lower concentrations of TNF-α, IL-1β, and MDA along with reduced apoptosis in the brain due to the regulation of Bcl-2 and Bax expression compared with the control group [24].

In research on microglia stimulated by LPS, it was found that the application of dexmedetomidine results in the upregulation of miR-340, one of the micro-RNAs that modulate inflammation [25]. Micro-RNAs are non-coding molecules that affect gene expression; they are involved in many biochemical processes, including oxidative stress, and they have been observed to play roles in Alzheimer’s disease, Parkinson’s disease, and Huntington’s disease [26].

Isoflurane and sevoflurane affect the cytokine concentrations in brain tissue, downregulating the expression of pro-apoptotic genes and upregulating the expression of anti-apoptotic genes. It has been shown that sevoflurane has a greater impact on the anti-apoptotic pathway [27]. However, isoflurane reduces neuronal apoptosis and inflammation while increasing the integrity of the blood–brain barrier through its impact on the activation of heme oxygenase-1. Brain magnetic resonance imaging (MRI) performed on septic mice receiving treatment with isoflurane revealed a lower diffusion of IgG in the perivascular regions, which indicates a reduction in blood–brain barrier disruption. In the brain cortex, increased cyclooxygenase-2 activity and changes in the size and shape of microglia were found in the corpus callosum [28,29].

## 3. Brain–Gut Axis

There is substantial evidence for communication between the brain and the diverse gut microbiota. Substances produced by the microbiota can serve as signals that are received by the brain and by the nervous and immune systems, which both mediate these relations. The dysregulation of the brain–gut axis and dysbiosis are observed in many conditions, especially in neurological and behavioral diseases [30]. The medication commonly used in sepsis treatment affects the homeostasis of the gut microbiome; the gut–blood barrier can be disrupted, allowing the substances that are produced by bacteria to easily cross the barrier, which contributes to sepsis severity [31].

### 3.1. Animal Studies

In research on rats that had received an intravenous injection of LPS from *E. coli*, a less diverse microbial composition was found in feces. The highest increase in bacterial load was presented by the phylum Proteobacteria, while Actinobacteria remained unchanged; the numbers of Firmicutes and Bacteroidetes were significantly reduced. After fecal microbial transplantation (FMT), the numbers of Firmicutes and Bacteroidetes increased, and the number of Proteobacteria decreased. Moreover, in a group of septic rats, the concentrations of TNF-α, IL-1β, and IL-6 were significantly higher, but they decreased after FMT. These results were compared with encephalogram records, and it was found that the group of rats that received FMT had a lower incidence of abnormal activity [32].

Different research studies have shown that mice that received a fecal microbiota transplant from mice with sepsis-associated encephalopathy who were then treated with antibiotics for five days all died within 48 h of treatment initiation. Sepsis was induced in this model via cecal ligation and puncture (CLP), and the mice with SAE were selected based on their neurological scores. Conversely, another group of mice that received FMT from healthy mice showed a 40% survival rate. It is presumed that the NLRP3 inflammasome plays a significant role in the development of SAE, as an analysis showed that there were increased levels of NLRP3 and IL-1β in the cortices of septic mice. The administration of indole-3-propionic acid (IPA), which is a metabolite of tryptophan produced by the gut microbiota, lowered the levels of both substances. The same results were obtained from an in vitro study. Mice that received IPA had longer survival times and better cognitive functions [33]. Another research study conducted on mice showed that the severity of sepsis and the damage to the hippocampus are related to the gut microbiota. Researchers divided septic mice (after CLP) into two groups, one with severe sepsis encephalopathy and another with mild sepsis encephalopathy. The distinction between severe and mild was made based on the survival term and neurobehavioral test scores. Feces from both groups were collected, and in the next step, they were transplanted into another group of mice after CLP. The results showed that the mice that had received feces from the mice with severe sepsis encephalopathy suffered greater damage to the hippocampus. To clarify the differences, fecal samples from both groups were analyzed in terms of the metabolites produced from the microbiome. There were many differences, but the most statistically significant difference was the change in butyric acid concentration, which was higher in the group of mice with a longer survival rate. Further analysis showed that butyric acid, produced by bacteria, reduces oxidative stress in the hippocampus and limits damage [34].

Research was conducted to explain the role of NU9056, an inhibitor of H4K16 histone acetyltransferase KAT5, which activates nitric oxide synthase and the acetylation of the inflammasome in SAE. This confirmed the possible involvement of short-chain fatty acids (SCFAs) in SAE pathology. It was observed that NU9056 increased the concentration of SCFAs in the feces of septic mice. Further research is needed to explain this discovery. The application of NU9056 ameliorated the SAE symptoms, stabilized the blood–brain barrier, and reduced oxidative stress [35].

The role of SCFAs was also investigated in another study. In mice, after 7 days of SCFA supplementation, CLP was performed; a higher expression of tight junction proteins, lower concentrations of IL-1β and IL-6 in the plasma, a lower expression of COX-2 and CD11b, and the phosphorylation of JNK inhibitor, NF-κB, and p65 were observed in the brains [36]. In septic mice, the levels of acetic and propionic acids, which are products of *Allobaculum*, *Bacteroides*, and *Bifidobacterium*, were measured. Both the bacterial and acid levels were decreased, and the mice showed cognitive impairments. Supplementation with SCFAs decreased the levels of proinflammatory cytokines and improved mental function. Moreover, to clarify the mechanisms of these changes, the mice were administered a GPR43 antagonist, GLPG0974, which reduced the neuroprotective effect [37]. GPR43 is a G protein-coupled receptor activated by SCFAs that occurs in many tissues, and it is suspected to be related to inflammatory diseases [38].

Research shows that probiotic supplementation has an impact on brain function. Healthy mice that were fed some strains of *Bifidobacterium* showed better learning ability and memory [39]. In research on septic mice treated with *Clostridium butyricum*, an improvement in the microbial balance in the gut was found, with decreased numbers of *Acinetobacter*, *Akkermansia*, *Eubacterium*, and *Escherichia-Shigella*. Moreover, brain-derived neurotrophic factor and proinflammatory cytokine levels were decreased. Additionally, the brain structure was found to be more organized in microscopic observations [40].

### 3.2. Human Studies

Human studies show that supplementation with *Lactobacillus plantarum* leads to lower levels of anxiety and stress and that septic patients who were treated with these bacteria presented better memory and cognitive functions. A lower concentration of proinflammatory cytokines in the plasma was also found [41]. Another study examining supplementation with *Bifidobacterium bifidum* and *Bifidobacterium longum* showed changes in the Korean version of the Consortium to Establish a Registry for Alzheimer’s Disease Assessment packet (CERAD-K) through a validated self-reported questionnaire after 12 weeks of supplementation compared with the placebo group. The most significant changes concerned the stress score, which was decreased in the probiotic group. Moreover, the serum levels of brain-derived neurotrophic factor (BDNF), which plays a role in neuroregeneration and neurogenesis, were found to be higher in the probiotic group [42]. Therefore, probiotic bacterial strains may be useful for alleviating SAE symptoms.

## 4. Statins

Statins are substances commonly used for cardiovascular protection, but the many years of use have demonstrated their multifunctional effects on human organisms. For this reason, they have become an object of research in terms of preventing SAE.

### 4.1. Animal Studies

In an animal study on rats treated with simvastatin after CLP, a decreased concentration of nitrate and IL-6 in the cerebrospinal fluid was observed. A similar decrease was not observed in the plasma. Moreover, in slides of the hippocampus and prefrontal cortex, a decreased level of aggregation of amyloid and Ser-360-tau protein concentration (both markers of neurodegeneration) and an increased concentration of Bcl-2 protein, which reduces neuronal apoptosis, were found. In addition, synaptophysin concentration, which can be a cause of behavioral disorders such as hyperactivity and the impairment of learning and memory activities, was at a higher level in the hippocampus of the sepsis survivor rat. The concentration in the prefrontal cortex stayed at a low level, which is a condition induced by sepsis. Protective effects against astrogliosis and higher microglia activation were found in both structures. Behavioral tests showed that rats treated with simvastatin had better memory, were more curious about new objects, and maintained better freezing reaction times [43].

A similar study carried out on the same model showed lower concentrations of nitrate, IL-1β, and IL-6 in the plasma, and it was found that simvastatin did not affect the TNF-α concentration in the plasma. The research also showed that simvastatin decreased the levels of thiobarbituric acid-reactive substances and increases citrate synthase and catalase activities in the hippocampus, leading to a reduction in oxidative stress. Simvastatin was found to increase mean arterial pressure (MAP), but the final heart rate (end-HR) and mortality did not differ between the groups, as shown in rats after conducting CLP. The same study showed that simvastatin reduces BBB permeability and brain edema. As an explanation, higher concentrations of the occlusive proteins claudin-3 and claudin-5 in rat brains were observed [44].

Another study was performed to elucidate the impacts of atorvastatin and simvastatin on septic mice, wherein models were prepared using i.p. fecal injections. The research indicated that treatment with statins did not change the acute mortality, which was 53%; however, statins were demonstrated to have positive effects in cognitive tests performed on septic survivor mice. Further biochemical tests showed that statins decreased the concentrations of chemokines (MPO-1 and KC), IL-1, and IL-6, and increased IL-10 concentrations in brain tissue compared with the control group. The same results were obtained for serum concentrations. Both statins reduced the concentrations of MDA and MPO in brain tissue, mitigating oxidative stress. Moreover, statins reduced the activity of Nox2 and increased NO endothelial expression, which prevents vasoconstriction in response to acetylcholine; in this case, the effect of atorvastatin occurred earlier—after 6 h—while simvastatin had an effect after 24 h, contributing to improved cerebral circulation [45].

Atorvastatin treatment administration before the CLP procedure increased the survival rate from 30% to 65%. The mice had better motor skills, less anxiety, and better results in the Morris water maze test. Moreover, atorvastatin reduces inflammation by decreasing the concentrations of IL-1, IL-4, IL-6, and TNF-α in the plasma, and it reduces oxidative stress by modulating SOD and CAT. A study on brain tissue found that there was a lower concentration of astrocytes in the cortex and a higher number of neurons in the hippocampus, which proved the anti-apoptotic impact of atorvastatin [46].

### 4.2. Human Studies

In a human study, Morandi et al. conducted research on ICU patients with sepsis and divided them into groups: those who had used statins before hospitalization and then had continued or stopped treatment at the ICU, non-statin users, and statin users at the ICU. Unfortunately, the research did not mention exactly which statins were used, only that different statins with simvastatin as the lead were used. Nevertheless, the results are interesting. The study showed that using statins before hospitalization did not have an impact on the incidence of delirium, which was 78% compared with 77%, but the group of patients who stopped treatment had a higher frequency of delirium, which was equal to 80%. When the therapy was continued, the incidence of delirium was the same as that in the non-statin group. In patients for whom a statin was included in the ICU, the incidence of delirium was lower, but it was related to the day of hospitalization and sepsis [47]. Another research has shown that patients treated at ICU with rosuvastatin had a lower incidence of SAE, which was 32% compared with 57% [48].

## 5. Hydrogen

Hydrogen is a potent antioxidant [49]. Thanks to its excellent action against oxidative stress, it has found wide applications in medicine. In all of the available research, it is agreed that treatment with molecular hydrogen ameliorates the physical and cognitive dysfunctions caused by sepsis in animal models. Researchers have indicated many mechanisms of influence.

### 5.1. Animal Studies

Research on septic mice has shown a connection between H_2_ and nuclear factor erythroid 2-related factor 2 (Nrf2) in terms of ameliorating sepsis and the symptoms of SAE. Nrf2 is a transcription factor that regulates the expression of antioxidant proteins. To clarify the connections between H_2_ and Nrf2, mice were divided into two groups: a wild type and a phenotype without Nrf2. Sepsis caused changes in microglia polarization in both groups. The effects of H_2_ only appeared in the wild-type mice. It was observed that inhaled H_2_ decreased the release of TNF-α, IL-1β, HMGB1, and NO. The inhalation of H_2_ induced the expression of Nrf2 in the cerebral cortex, consequently decreasing the expression of LDH, caspase3, and Bax/Blc-2, which are involved in the apoptotic pathways that are regulated by Nrf2, thus protecting neurons from damage [50]. Researchers also observed an effect on the stabilization of the blood–brain barrier via increased levels of adhesive proteins [51]. Other studies explained that H_2_ limits the activation of the NLRP3 inflammasome via the Nrf2 pathway, which causes a decreased concentration of proinflammatory cytokines [52]. The same progress in behavioral tests and changes in cytokine concentrations in septic mice inhaling H_2_ were observed in studies in China. Moreover, the authors found increases in the concentrations of IL-10 and TGF-β. IL-10 is a cytokine synthesis inhibitory factor, and transforming growth factor beta (TGF-β) is a substance that controls the proliferation and differentiation of cells; both lead to a reduction in inflammation. The authors indicated that the reason for this is the inhibition of mTOR kinase; this inhibition promotes neuronal autophagy and modulates microglia polarization [53]. One study chose to analyze the impact of H_2_ inhalation on the gut microbiome of mice because it is the main gas in the intestines. Molecular hydrogen affects microbial metabolism and transmission in the gut–brain axis. As such, the authors suspected that changes in H_2_ concentration may have affected the course of sepsis. The intestinal microflora were the most greatly changed over 24 h after CLP processing, the same as with brain function, so the experiment was performed at that time. The results showed that the inhalation of molecular hydrogen and drinking of hydrogen-rich water increased the survival rate of septic mice. The expression of TNF-α, IL-6, and HMGB-1 in SAE mice was increased, but in terms of brain homogenates, the levels were lower in the molecular hydrogen-treated mice. In the collected feces, it was found that treatment with H_2_ changed the composition of the gut microbiome, but most of the results were not statistically significant. Nevertheless, decreased levels of pathogenic bacteria were observed. The levels of metabolic biomarkers in the brain, such as pantothenate, *N*-acetylneuraminic acid, sarcosine, tryptophan, and DL-serine, were higher in the molecular hydrogen-treated mice, which can lead to an improvement in the stability of the blood–brain barrier and brain homeostasis [54].

In their research, Bai, Y. et al. noticed that the treatment of septic mice with H_2_ increased the survival rate from 40% to 60% on day 7. To explain this change, the authors analyzed brain tissue and found that pyramidal neurons in the hippocampal area were damaged in the CLP group, but that the destruction was reduced in the CLP + H_2_ group, with most neurons having a proper structure. Moreover, they observed a higher number of Nissl bodies and a reduction in apoptosis in the CLP + H_2_ group compared with the CLP and sham groups. Further analysis showed changes in protein phosphorylation in the H_2_-treated group, especially in the case of serine, which regulates cell functions. The authors postulated that the PI3K/Akt signaling pathway mediated this process [55].

In a group of rats treated with hydrogen-rich saline (HRS) 48 h after CLP processing, the survival rate was 81% compared with 25% in the LPS group. As in the previous research, increased numbers of normal neurons and Nissl bodies in the cerebral cortex were found in the HRS group. Neuronal apoptosis was lower, which later confirmed the decrease in Bax expression and increase in Blc-2 expression. The ELISA assay showed a decreased concentration of TNF-α and IL-1β and an increase in Li10 in response to the HRS, and it was most visible after 1 h of sepsis induction. The HRS group also presented reduced activations of astrocytes and microglia and lower mitochondrial dysfunction and damage. The concentrations of markers of neuronal injury, such as GFAP and Iba-1, were lower after HRS treatment [56].

In septic mice, mitochondrial dysfunction was observed, and research was conducted to clarify the reason for this. It was found that treatment with H_2_ increased the mitochondrial membrane potential, ATP amount, and activity of mitochondrial respiration chain complex I, but it did not affect the decreased activity of mitochondrial respiration chain complex II. In addition, hydrogen alleviated mitochondrial swelling and the disappearance of ridges caused by sepsis. The expression of molecules that play a role in mitochondrial biogenesis, such as PGC-1α, NRF2, and Tfam in the hippocampus, was increased after hydrogen treatment. To determine the mechanism underlying the impact on the mitochondria, SR-18292, an inhibitor of PGC-1α, was used, which stopped the positive impact of H_2_. Therefore, PGC-1α is considered to play an inseparable role in mitochondrial protection [57].

It was predicted that H_2_ inhalation would affect the methylation of DNA. In the hippocampi of sepsis mice treated with molecular hydrogen inhalation, decreased levels of mRNA for DNA methyltransferases, such as DMT1 and DMT3a, were found; these are able to induce changes in behavior or memory by affecting DNA methylation. Moreover, a higher expression of brain-derived neurotrophic factor (BDNF), one of the neuronal growth factors, was observed due to the modified methylation of its promoter [58]. Another study on the hippocampi of mice showed a decreased level of phosphorylated tau protein and an improvement in cognitive functions after H_2_ inhalation [59].

### 5.2. Human Studies

To date, no clinical human studies have been performed using hydrogen with respect to sepsis; however, a study was conducted in China on humans suffering from COPD. Hydrogen/Oxygen therapy was applied at a volume ratio of 2:1 and flow rate of 3 L/min, which obtained very promising results in terms of the amelioration of COPD symptoms. More importantly, this study confirmed that hydrogen can be safely used in human therapy [60].

## 6. Drugs Acting on the Nervous System

Neuroglobin (Ngb) is known as a protein that improves brain oxygenation via its ability to bind oxygen, which is why it is used to ameliorate hypoxic and ischemic effects [61]. It has also been investigated for use in treating SAE due to these properties. Zhang et al. explained how the expression of neuroglobin impacts SAE symptoms. The authors induced or blocked neuroglobin expression using hemin in one group of animals and an antisense nucleotide in the other. It was found that, in the hippocampus and frontal cortex, increased neuroglobin expression protected mitochondria from damage and limited apoptosis, as detected using a TUNEL assay [62]. Another study indicated that the level of neuroglobin significantly rose 24 h after CLP and returned to baseline after 48 h. Neuroglobin ameliorated structural damage and the edema of neurons. The examined rats had better results in the Morris water maze test; however, pretreatment with the PI3K/Akt pathway inhibitor LY294002 limited this effect. The levels of Bax and Blc-2, which are part of the PI3K/Akt pathway, were measured. Increased expression of Ngb reduced the Bax level but did not impact Blc-2 levels. Moreover, LY294002 counteracted the changes in Bax concentration without affecting Blc-2 [63].

Selegiline, a monoamine oxidase inhibitor that has proven therapeutic effects in neurodegenerative diseases, improved the blood–brain barrier’s stability by increasing the expression of tight junction proteins and by reducing the concentrations of proinflammatory cytokines, according to a research study conducted on mouse brains [64]. Studies on humans in the context of alleviating sepsis symptoms have not been performed, but one study was conducted on a group of patients with prolonged disorder of consciousness who were assessed using the Coma Recovery Scale-Revised (CRS-R), which indicated that selegiline helped with the recovery process. However, the limitation of this study is the small size of the study group [65].

Valproic acid, which is routinely used in epilepsy treatment and some psychiatric diseases, activates the BDNF–TrkB signaling pathway in the brain and ameliorates cognitive dysfunction, reducing neuroinflammation and the apoptosis of neurons [66].

Stanniocalcin is a protein that binds to the cell surface in many tissues, including the mitochondrial membrane, and it plays a role in inhibiting oxidative stress. In vitro research on microglia stimulated by LPS and cultured with recombinant human stanniocalcin (rhSTC-1) demonstrated a decrease in the production of proinflammatory cytokines and better microglial viability. In the hippocampi of rats after CLP, a decrease in the activity of creatine kinase was observed, which was increased after rhSTC-1 administration. Moreover, rhSTC-1 increased the activities of complexes 1 and 2, and these were limited by sepsis. The elevated nitrate concentration in the hippocampus observed in the septic group was ameliorated by rhSTC-1, which proves its antioxidative effect. Finally, an improvement in memory was noticed in the sepsis survival group of rats treated with rh-STC-1 [67].

## 7. Antibiotics

Pentamidine, a medication used in leishmaniasis, lowered the expression of FAP, S100B, RAGE, and NF-κB in the hippocampus. As a consequence, it reduced oxidative stress and decreased the concentrations of TNF-α, IL-1β, and IL-6, according to a research that used mouse models [68]. The research indicated that minocycline, a tetracycline antibiotic medication, protected hippocampal long-term potentiation (LTP) in sepsis mice. LTP is a process that creates stronger connections between neurons, as was the case in the study, so the impairment of LTP could be connected to cognitive deficits after sepsis [69]. It has become apparent that many substances of natural origin can have neuroprotective effects in the treatment of SAE.

## 8. Plant-Derived Drugs

The Chinese plant *Ligusticum chuanxiong* is used as a sleeping aid. The substance that is isolated from it is named senkyunolide I. Research on septic mice treated with senkyunolide I showed a decrease in the concentrations of TNF-α, IL-1β, and IL-6 in the plasma and better results in cognitive tests [70]. Probenecid, a substance commonly used in the treatment of gout and hyperuricemia, can inhibit pannexin-1, which is a non-selective ion-gated channel protein that is involved in the induction of pyroptosis. Research has proven that blocking this channel ameliorates the symptoms of sepsis-associated encephalopathy [71]. Fisetin, a color agent of many plants, can also play a role in many mechanisms that ameliorate SAE symptoms. One mechanism is the inhibition of neuroinflammation by modulating the expression of IL-1R1, pNF-κB, TNF-α, and iNOS in microglia as well as by affecting the induction of mitophagy [72]. Morin is a flavone that exists in many natural products. In septic mouse models, it was found to decrease the expression of IL-6, MCP-1, TNF-α, and IL-10 in the serum. Moreover, it reduces microglia activation, decreases tau phosphorylation, and reduces amyloid deposition [73]. Ginseng contains a substance named ginsenoside Rg1 (Rg1), which has been used in the treatment of septic mice. It increased the survival rate from 60% to 80% at 7 days after CLP processing and improved the cognitive function of the mice. Further examinations showed reduced neuroinflammation and neuronal apoptosis [74,75]. Caffeine citrate, which is commonly used by neonatologists to treat apnea of prematurity, can also play a neuroprotective role in SAE; it is able to inhibit neuronal apoptosis and neuroinflammation via the UPC2/NLRP3 axis in astrocytes [76,77]. A deficiency in acetylcholine is a probable cause of SAE. Researchers thus aimed to elucidate the impact of huperzine A on SAE; huperzine A is an acetylcholinesterase inhibitor. In the hippocampi of rats suffering from SAE, after huperzine A treatment, a higher concentration of acetylcholine was found compared with that observed in the group without treatment. Moreover, an increased expression of the genes for choline acetyltransferase and muscarinic acetylcholine receptor-1 was observed. The levels of proinflammatory cytokines were lower in the huperzine A group, and neuronal apoptosis was less. After treatment, the rats achieved better results in the Morris water maze test [78]. According to in vitro and in vivo research on LPS-induced sepsis mouse models, borneol, an ingredient in many essential oils, had an inhibitory effect on the MAPK and NF-κB signaling pathways. As a consequence, microglia activation decreased [79]. Butein, a pigment found in many plants that are yellow in color, enhanced SIRT-1 signaling. This improved the mortality rate, lowered brain edema, and decreased proinflammatory cytokine concentrations and neuronal apoptosis [80].

## 9. Other Molecules

Cortistatin-14 is an endogenous peptide that can bind to somatostatin receptors, but it also has different functions, such as modulating sleep and reducing locomotor activity. The expression of cortistatin is especially high in the cortex, where it decreases neuronal activity in a process that is similar to that which occurs for somatostatin [81]. In mice, the level of cortistatin was decreased after CLP. After initiating treatment with cortistatin, higher survival rates, decreased levels of proinflammatory cytokines, lower microglia activation, and better blood–brain barrier integrity were observed [82]. During the in vivo PET brain imaging of septic mice, a decreased rate of glucose metabolism was found. Imaging was performed with and without fructose-1,6-bisphosphate treatment. After treatment, the mice had a similar rate of glucose metabolism to healthy mice. Moreover, an increase in the activity of catalase (CAT), which reduces oxidative stress, and a higher concentration of S100B protein were found [83]. Research on some commonly used drugs has shown that they can play neuroprotective roles in the treatment of SAE. One of these drugs is the tricyclic antidepressant amitriptyline, which limited neuronal loss in the hippocampus via the TrkA pathway and limited microglia activation, as indicated in research performed on mice after CLP processing. The neuroprotective effect was confirmed using the Morris water maze test. The researchers also noticed lower levels of oxidative stress [84].

Secukinumab is a monoclonal antibody against interleukin-17 A that decreased cognitive impairment in a group of septic rats, and it reduced oxidative stress and neuronal apoptosis [85]. Rapamycin, a macrolide antibiotic with immunosuppressive effects, alleviated cognitive impairment in septic rats. In the septic group treated with rapamycin, lower levels of phosphorylated mTOR and phosphorylated AKT were found; these are typically inhibited by rapamycin. The levels of phosphorylated p70S6K also decreased. Consequently, neuronal autophagy was blocked, and cognitive functions were rescued [86]. Similar results were obtained when studying the effects of erythropoietin, an endogenous hormone [87]. Moreover, researchers found that JAK2/STAT3 signaling could be involved in the process in the case of erythropoietin [88].

Metformin administered to SAE rats lowered the activity of HMGB1, a component of DAMPs. This resulted in decreased inflammation. Additionally, the concentrations of S100B, neuron-specific enolase, and glial fibrillary acidic protein decreased. The expressions of claudin-3 and claudin-5 were higher, which stabilized the blood–brain barrier. Clinically positive changes in the Murine Sepsis Score were observed [89].

## 10. Conclusions

**Figure 1:**
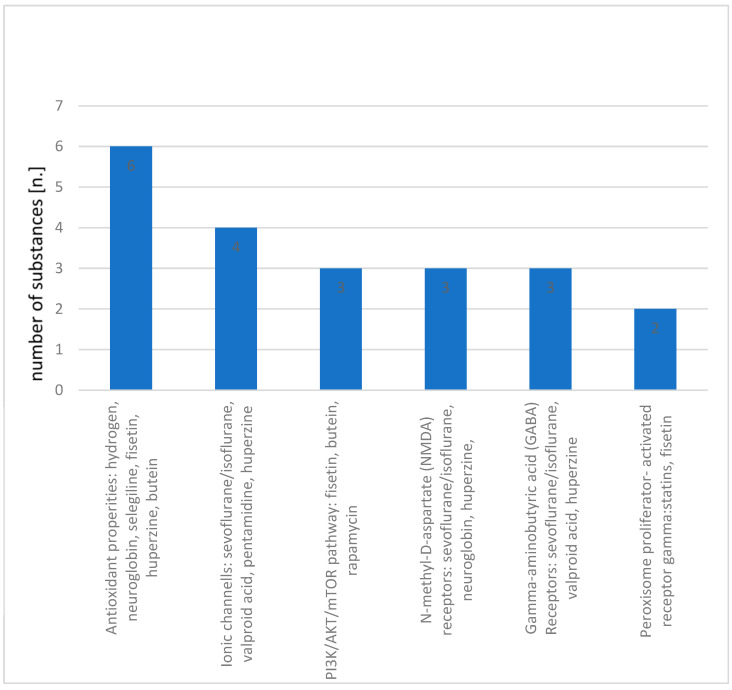
The most commonly repeated mechanisms of action of neuroprotective substances.

Sepsis-associated encephalopathy is a complex and multifaceted condition that requires a targeted and comprehensive approach to treatment. The numerous substances that have been identified in research as potential treatments for SAE highlight the diverse mechanisms by which this condition can be addressed. While it is promising that combining multiple substances with different mechanisms of action could increase the neuroprotective effects, there is a clear need for further research in this area. For better clarity, the mechanisms of operation have been organized in Table 2 and Figure 1.

To date, only a few of the substances identified from the research have been tested on humans, and it remains to be seen whether they can be safely and effectively used for treating SAE within clinical settings. Therefore, it is imperative that further research is conducted in order to better understand the efficacy and safety of these substances in treating SAE.

In addition to identifying new treatments, there is also a need to establish clear diagnostic criteria for SAE. This will allow for the early and accurate detection of the condition, which is crucial for successful treatment. More research is also needed to establish the prevalence of SAE and its impact on long-term patient outcomes.

Overall, the research into potential treatments for sepsis-associated encephalopathy has identified several promising substances with diverse mechanisms of action. However, further research is needed to establish their safety and efficacy in treating SAE in humans. Additionally, more work is needed to establish diagnostic criteria, determine the prevalence of SAE, and understand the impact of the condition on long-term patient outcomes.

## Figures and Tables

**Table 1:** Characteristics of sepsis-associated encephalopathy.

Clinical Features of SAE	Deterioration of Communication, Confusion, Drowsiness, Agitation, Seizures, Hallucinations, Coma
Epidemiology of SAE	9–70% of patients with sepsis
Prognoses	Increased mortality rate up to 60%
Long-term consequences	Symptoms similar to dementia

**Table 2:** Mechanism of action of the most promising neuroprotective substances.

Dexmedetomidyne	Highly selective alpha-2 adrenoceptor agonist
Sevoflurane, isofluran	Enhance the activity of the gamma-aminobutyric acid (GABA) and *N*-methyl-d-aspartate (NMDA) receptors
Hydrogen	Antioxidant properties, regulation of intestinal microbiome, regulation of gene expression
Neuroglobin	Oxygen binding and transport, antioxidant properties, regulation of gene expression
Selegiline	Inhibition of MAO-B, antioxidant properties
Cortistatin	Depression of neuronal activity and inhibition of cell proliferation, binds to somatostatin receptors
Metformin	Lowers the activity of high mobility group box 1 (HMGB1)

## Data Availability

No new data were created or analyzed in this study. Data sharing is not applicable to this article.
